# Research on an On-Chip MEMS Based Safety and Arming Device with a Mechanical Encryption System

**DOI:** 10.3390/mi10060407

**Published:** 2019-06-19

**Authors:** Kuang Fang, Tengjiang Hu, Xueting Zhang, Zhiming Zhang, Xiaohua Jiang, Yulong Zhao

**Affiliations:** 1 School of Mechanical Engineering, Xi’an Jiaotong University, Xi’an 710049, China; kuangf302@126.com (K.F.); htj047@xjtu.edu.cn (T.H.); xueting_z@126.com (X.Z.); 2 Institute of Chemical Material, China Academy of Engineering Physics, Mianyang 621900, China; 13330881511@189.cn

**Keywords:** Safety and Arming Device, encryption system, chevron actuator

## Abstract

The design and characterization of microelectromechanical systems (MEMS) based on-chip SAD (Safety and Arming Device) are proposed. An encryption system has been integrated into the device to enhance its reliability during the electromagnetic interference. The conversion between safe status and arm status is reversible due to the bidirectional actuation design of the slider and pawl on the SOI (Silicon on Insulator) chip, being driven by the chevron electrothermal actuators. The width of each tooth on the slider, which contains coding information, is different from that of its adjacent neighbor. Additionally, the different teeth width, respectively 32 μm, 82 μm, requiring different decoding displacement of 100 μm and 150 μm, corresponds to the different decoding voltage of 13.5 V and 14.8 V. The travel range of interrupter in the SAD will only be limited by the chip dimension and be able to cover the motion of ±1 mm in the present research, due to the capability of motion retention. Finally, the SAD is integrated with a copper azide exploding chip to measure the average velocity of the titanium flyer for the application feasibility validation.

## 1. Introduction

The Safety and Arming Device (SAD) is the essential component that prevents fuze arming until specific conditions have been achieved [1]. The core principle of safe and arm mechanism is the interruption of energy transfer, usually explosion, laser, or high-speed flyer, with a movable interrupter. The safe and arm function need to be carried out in a very tiny volume and operate with a high reliability level due to the development tendency of munition system miniaturization. The difficulty that we faced is not only how to achieve sufficient displacement or force output for the interrupter in the limited space [2], but also how to guarantee the safe and arm function in the complicated environment of battle field. 

Microelectromechanical systems (MEMS) technologies demonstrate profound potential to tackle the paradox that we faced, particularly, mems actuators and their peripheral mechanical structures, such as multiple levers, pivots, and springs, applied in the micro robot, scanning probe microscopy, and micro optical lens scanner [3,4,5]. The safe and arm mechanism should function aligning to proper amount of electrical energy input to satisfy the need of accurate motion control for SAD. Owing to the advantages of low operating voltage, lateral motion parallel with the substrate plane, and the reliable deflection and force output [6,7], the electrothermal strategy is widely applied in the SAD [8,9,10]. While, in the most of the previous researches on the MEMS based SAD [2,6,7,8,9,10], the deformation of mems actuator, usually no more than 500μm, limits the displacement of interrupters, and all kinds of device are controlled by the simple electrical signal, which makes the devices vulnerable under the high intensity electromagnetic interference.

In this research, we propose the prototype of an on-chip SAD that is based on the chevron electrothermal actuators. Due to the feature of motion retention, the travel range of interrupter in the SAD will only be limited by the chip dimension. To enhance the reliability during the electromagnetic interference, a unique mechanical encryption system has been integrated into the SAD. Different from our previous researches [11], the chip could only be disarmed through a unique amplitude sequence of the input voltage; otherwise, it will be stuck or locked down by the interlock mechanism. 

## 2. System Composition 

### 2.1. Device Description

**Figure 1:**
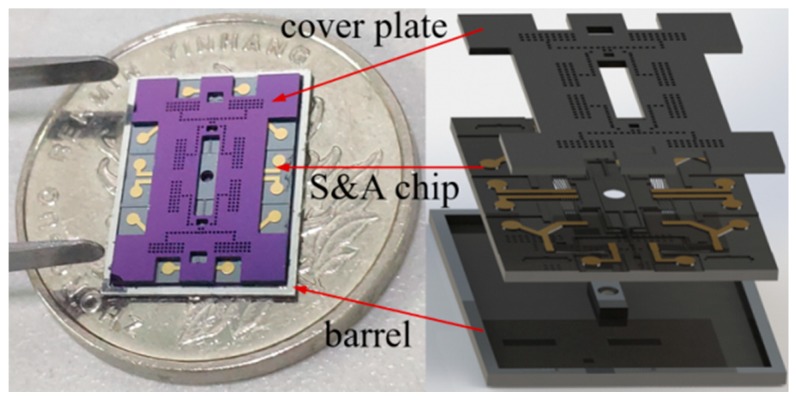
The structure of on-chip microelectromechanical systems (MEMS) based Safety and Arming Device (SAD).

The on-chip SAD consists of a cover plate, the silicon barrel, and the S&A (safety and arming) chip, as shown in Figure 1. The cover plate just acts as a protective maskant. The barrel serves to shear a portion of foil, driving by the explosion of micro-charge, under the SAD, and it acts as a channel for the detached flyer on its way to detonate the high explosive pellet.

**Figure 2:**
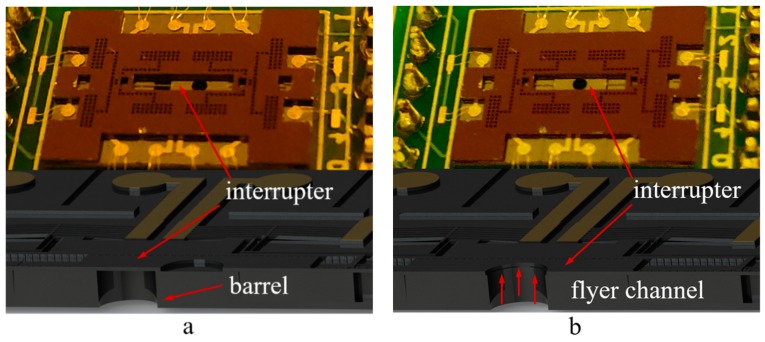
The conversion of the S&A (safety and arming) chip between safe status and arm status: (**a**) Safe status; and, (**b**) Arm status.

The S&A chip is the essence of the device, which is fabricated on the SOI (Silicon on Insulator) wafer with a dimension of 14.2 mm × 10.5 mm × 0.453 mm. As shown in Figure 2, with the interrupter that is driven by the chevron electrothermal actuators bidirectionally, the chip can convert between the safe status (Figure 2a) and the arm status (Figure 2b) to control the energy transfer in the explosive train.

**Figure 3:**
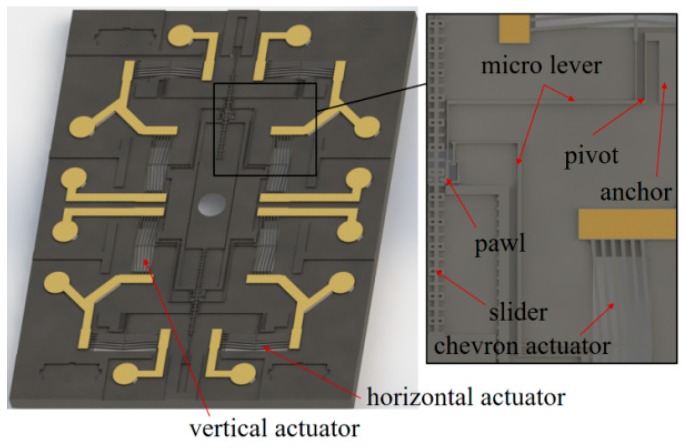
The structure of S&A chip. The interrupter is driving by the relative movement between the pawl and each tooth on the slider.

Four pairs of actuators are arranged axisymmetrically on the S&A chip with micro levers, pivots, and anchors to realize the bidirectional actuation. The micro levers and the micro slider are introduced to balance the force and displacement output of actuator, as shown in Figure 3. The displacement of interrupter can be divided into several steps, each of the step is a reflection of relative movement between the pawl and teeth on the slider. The maximum travel range of interrupter will only be limited by the chip dimension due to the capability of motion retention, as long as the input signal matches the driving requirement of each tooth on the slider.

As shown in Figure 3, the vertical actuators are designed for the disengagement/reengagement control between the pawl and slider, and the horizontal actuators serve for slider pulling. In addition, the leverage ratio of the micro lever is set to 20 to magnify the displacement output, within the proper consumption of pulling force. 

**Figure 4:**
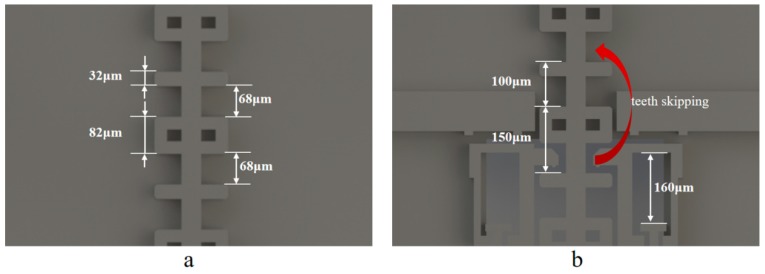
The illustration of the encryption system: (**a**) The slider with a unique sequence of teeth width; and, (**b**) The design for teeth skipping prevention.

In consideration of countering the hostile electromagnetic interference in the battle field, the encryption system on the S&A chip consists of a slider, the teeth width of which constitutes a unique sequence, and the pawls with the specific design for the teeth skipping prevention. The unique sequence of teeth width is a periodic repetition of 32 μm and 82 μm, with a 68 μm interval on the slider, as shown in Figure 4a. In order to prevent the miss decoding that is caused by teeth skipping, the maximum pawl movement is confined in 160 μm, when considering that teeth skipping would consume a relative movement of 250 μm at least, as shown in Figure 4b.

**Figure 5:**
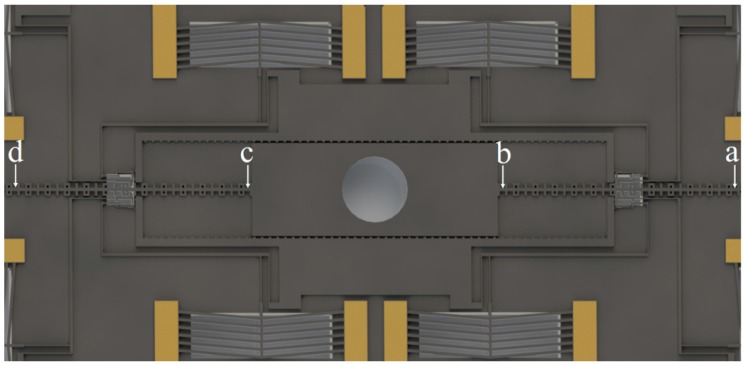
The illustration of teeth width sequence on the both sides of interrupter.

The teeth width sequence on the both sides of interrupter should be identical in the motion direction, specifically, the teeth width sequence on the slider from point c to point d, should be a duplication of that sequence form point a to point b, according to the requirement of bidirectional actuation and the guarantee for the reliability of disengagement/reengagement process, as shown in Figure 5.

### 2.2. Working Principle

When considering that there is a one-to-one correspondence between the input voltage and the output, force, or displacement, of electrothermal actuator, a specific width of the teeth on the slider should correspond to a unique decoding voltage for the pawl actuation, which forms the core mechanism of our encryption system. Accordingly, the arming process will be stuck if the decoding voltage is inaccurate. 

**Figure 6:**
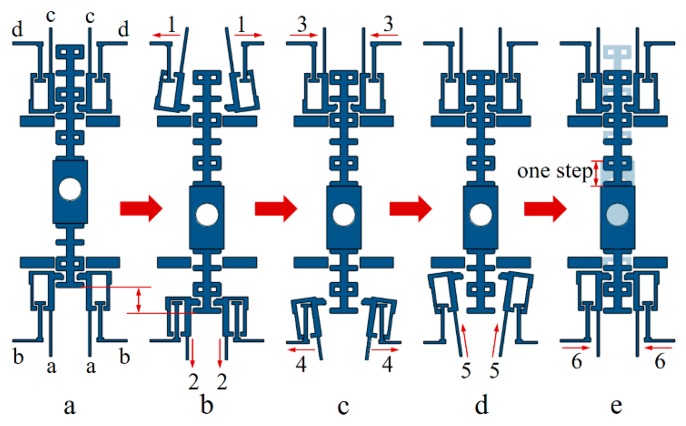
The operation process of S&A chip: (**a**) Initial state, the actuators are divided into four groups; (**b**) Step1, disengagement of the upper pawls and slider, and Step2, the slider is pulled by the lower pawls; (**c**) Step3, reengagement of the upper pawls and slider, Step4, disengagement of the lower pawls and slider; (**d**) Step5, the lower pawls realign with the slider; and (**e**) Step6, reengagement of the lower pawls and the slider.

For the clear instruction of arming and disarming process, eight chevron actuators combining with micro levers can be split into four groups, such as the vertical actuator groups (b, d) and the horizontal actuator groups (a, c), as shown in Figure 6a. Furthermore, one step movement of the interrupter can be divided into six substeps that are driven by the electrothermal actuator: Step1, 13 V voltages apply on the vertical actuator group d to disengage the upper pawls and the slider, as shown in Figure 6b; Step2, 14.8 V voltages apply on the group a to pull down the slider with a displacement of 150 μm, as shown in Figure 6b; Step3, the voltages on group d will be removed to reengage the upper pawls and the slider, meanwhile the position of interrupter will be locked, as shown in Figure 6c; Step 4, With the applied voltage holding on group a, 13 V voltages will be applied on group b to disengage the lower pawls and the slider, as shown in Figure 6c; Step5, the voltages that are applied on the group a are removed, and the lower pawls will realign with the interval between the teeth, as shown in Figure 6d; Step6, the voltages applied on the group b will be removed to reengage the lower pawls and the slider, four group of actuators will also return to their initial state.

**Figure 7:**
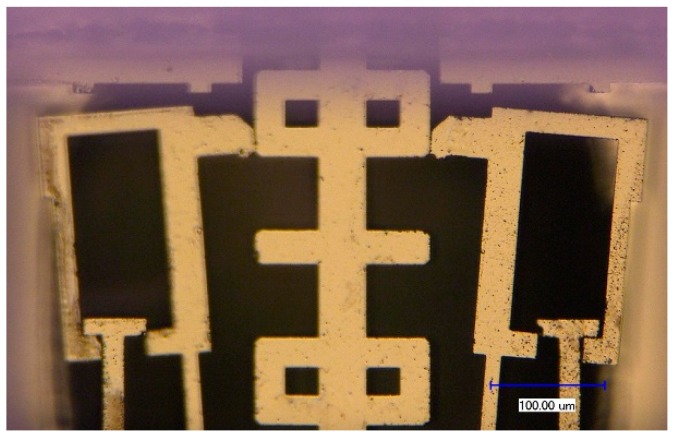
The pawls are stuck with the slider due to the inaccurate voltage input.

The process that is illustrated in Figure 6 specifically indicates a step of movement corresponding to the teeth on the slider with the width of 82 μm. For the teeth whose width is 32 μm, the pulling voltages in Step2, as shown in Figure 6b, will decrease to 13.5 V. It is obvious that each decoding step of arming/disarming processes must endure the recognizing by not only the voltage timing sequence of actuation signal, but also the amplitude of actuation voltage. With the inaccurate decoding voltage, the S&A chip will be stuck, as shown in Figure 7, or burnt out that is caused by overloading, under which circumstance the interrupter will be locked down.

**Figure 8:**
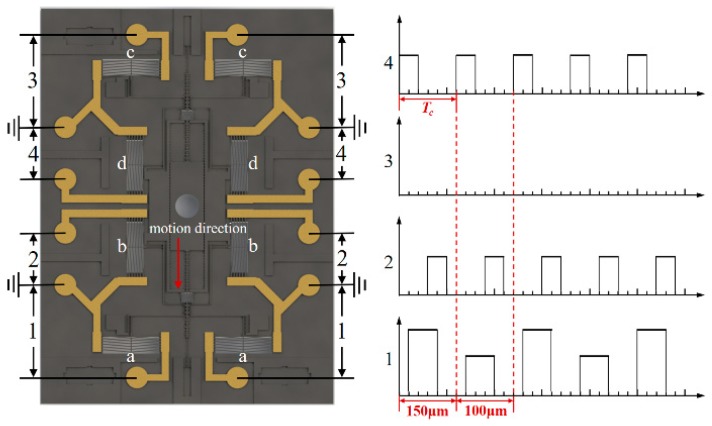
The driven signal of the pawls on the chip.

To approach a clear understanding of the voltage timing sequence that is applied on the S&A chip, the signal input on the bonding pads, during the disarming process, are split into four groups, according to the four groups of electrothermal actuators, as shown in Figure 8. Four groups of signals input on the bonding pads follow the same time line. The cycle time of each motion step of interrupter equals to $T_{c}$, whether the longer step, 150 μm or the shorter one, 100 μm. The core mechanism of our encryption system is the firm correlation between signal 1 and the unique sequence of teeth width on the slider in the motion direction, as shown in Figure 5. Meanwhile, considering the response time of electrothermal actuator usually lies in several milliseconds [12,13,14], the cycle time $T_{c}$ of 100 ms, in the present research, will be sufficient enough for the S&A chip to accomplish the one step movement of interrupter. In addition, the inverted actuation of interrupter can be achieved by swapping signal 1, 3 and signal 2, 4.

## 3. Theoretical Analysis

**Figure 9:**
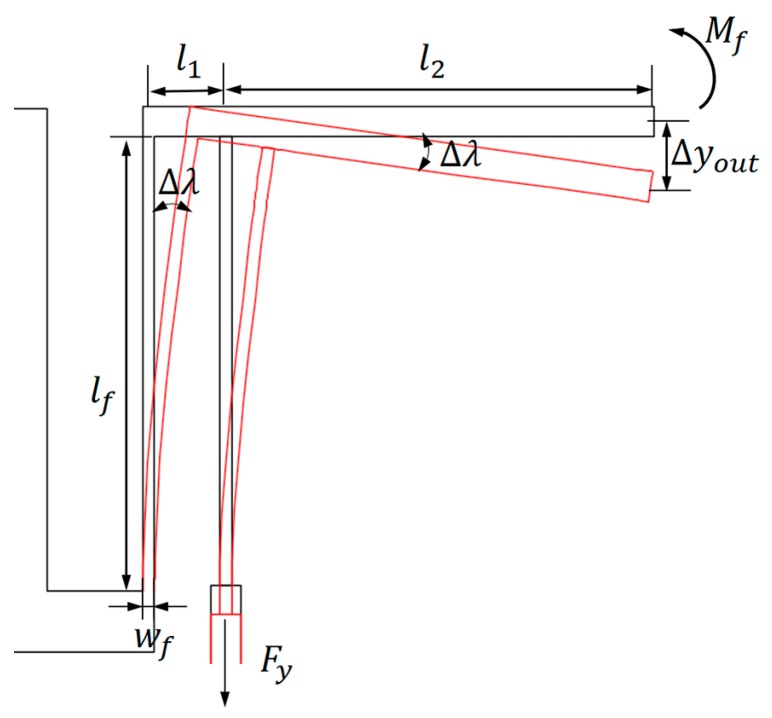
The micro lever for the displacement magnification.

**Figure 10:**
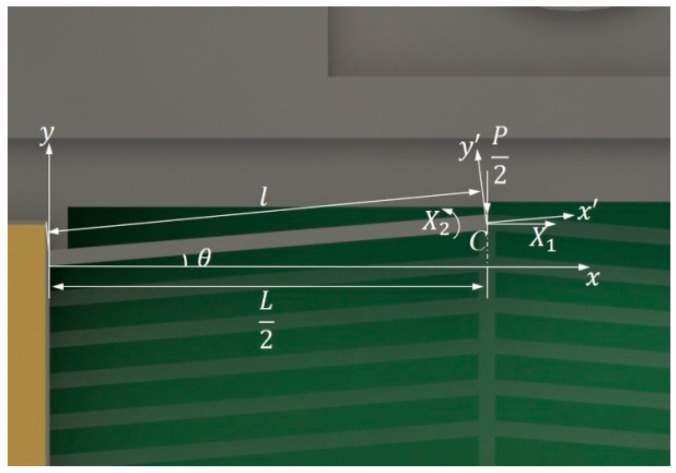
The V-shape beam in the chevron actuator.

The design of the encryption system is directly related to displacement output of the pawl in the horizontal actuator groups (a, c), as shown in Figure 8, which is dominated by the amplitude of voltages in signal 1, 3. We divide the horizontal actuator into two study objects, the micro lever (Figure 9) and the electrothermal chevron actuator (Figure 10) to analyze the relation between the pawl output and the voltage.

According to Xiao-Ping S. Sua and H.S. Yang [15,16], the un-anchored ends of the pivot beam and the connection beam connecting the output system and the lever arm will maintain a 90° orientation with respect to the lever arm after loading. In other words, the un-anchored ends of the pivot beam and the connection beam will both be rotated by the same angle Δλ at their respective joints with the lever arm. The micro lever in the present work, as shown in Figure 10, can be defined as the third-kind micro lever [15], the displacement output of the pawl in the motion direction of slider, $\Delta y_{\mathrm{out}}$, can be described as:

(1) $$\Delta y_{\mathrm{out}}=\left(l_{1}+l_{2}\right)\sin\Delta \lambda +\Delta y$$

The bending angle Δλ and the deformation along the flexible beam Δy can be obtained by the equation:

(2) $$\{\begin{array}{c} \Delta \lambda =\frac{(F_{y}l_{1}-F_{f}l_{3})l_{f}}{EI_{f}}\\ \Delta y=\frac{\left(F_{y}-F_{f}\right)l_{f}}{Etw_{f}} \end{array}$$

The parameter E is the Young’s modulus of silicon. $F_{y}$ is output force of the chevron actuator. $M_{f}=F_{f}\cdot l_{3}$ represents the rotation moment derived from the reactive force on the pawl, which is caused by the retarding force on the interface between the slider and substrate, according to the microtribology. $l_{f}$ and $w_{f}$ represent the length and width of the flexible beam, respectively. $I_{f}=tw_{f}^{3}/12$ is the moment of inertia. t is thickness of the flexure hinge. 

The analysis of the chevron actuator output force $F_{y}$ can be simplified as a statically indeterminate problem of a double-clamped V-shape beam, as shown in Figure 10. 

When considering the chevron actuator is symmetric with respect to cross section C, we just take the left half as our study object. $X_{1}$ and $X_{2}$ are the internal force and the bending moment produced by thermal expansion on the cross section C, respectively. The internal force of V-shape beam, reactive force from the micro lever, the deformation compatibility condition is that the horizontal displacement and the rotation angle on the section C are both equal to 0 due to the feature of axial symmetry, and under the effect of thermal expansion. Combing with principle of the force method of the structural mechanics, we can obtain following equations:

(3) $$\{\begin{array}{c} \Delta l_{t}\cdot \cos\theta +\delta_{11}X_{1}+\delta_{12}X_{2}+\delta_{1p}\cdot \frac{P}{2}=0\\ \delta_{21}X_{1}+\delta_{22}X_{2}+\delta_{2p}\cdot \frac{P}{2}=0 \end{array}$$

Here, $\Delta l_{t}=\alpha \cdot \Delta T\cdot l$ is the deformation that is caused by thermal expansion on the beam. α represents the coefficient of thermal expansion of monocrystalline silicon. $\Delta T=\int_{0}^{L}T\left(x\right)dx/L-T_{r}$ represents the average temperature change, $T_{r}$ is the reference temperature. $\delta_{ij}\left(i=1,2;j=1,2,p\right)$ represents the flexibility coefficient. $P=F_{y}/6$ is the component of reactive force from the micro lever on the V-shape beam. Bringing the expressions of $\delta_{ij}$ into Equation (3), $X_{1}$ and $X_{2}$ can be solved:

(4) $$\{\begin{array}{c} X_{1}=\frac{-12EAI\alpha \cdot \Delta T\cdot \cos\theta }{J}-\frac{\left(PAl^{2}-12PI\right)\sin\theta \cos\theta }{2J}\\ X_{2}=\frac{-6EAIl\alpha \cdot \Delta T\cdot \sin\theta \cos\theta }{J}+\frac{3PIl\cos\theta }{J}\\ J=Al^{2}\cdot \sin^{2}\theta +12I\cdot \cos^{2}\theta \end{array}$$

Here A=w·t is the cross-sectional area, w represents width of the beam, and $I=tw^{3}/12$ is the moment of inertia. Additionally, we establish a $x^{\prime }y^{\prime }$ coordinate system along the beam to simplify the calculation, as shown in Figure 10. The deformation on the axes are given by the equation:

(5) $$\{\begin{array}{c} \Delta x^{\prime }=\frac{X_{1}\cdot l\cdot \cos\theta }{EA}-\frac{P\cdot l\cdot \sin\theta }{2EA}+\alpha \cdot l\cdot \Delta T\\ \Delta y^{\prime }=\frac{X_{2}\cdot l^{2}}{2EI}-\frac{X_{1}\cdot l^{3}\cdot \sin\theta }{3EI}-\frac{P\cdot l^{3}\cdot \sin\theta }{6EI} \end{array}$$

When considering that the maximum vertical displacement $\Delta y_{\max}$ is reached under the condition P=0, $\Delta y_{\max}$ can be expressed as:

(6) $$\Delta y_{\max}=\Delta x^{\prime }\cdot \sin\theta +\Delta y^{\prime }\cdot \cos\theta =\frac{\alpha \cdot l\cdot \Delta T\cdot \sin\theta }{{\left(\frac{w}{l}\right)}^{2}+\sin^{2}\theta }$$

According to the one-dimensional (1-D) models of heat generation and dissipation [17,18], the temperature distribution on the V-shape beam can be solved, with thermal boundary condition: $T\left(0\right)=T\left(L\right)=T_{r}$. Therefore, we can obtain the expression of ΔT:

(7) $$\{\begin{array}{c} \Delta T=\frac{V^{2}\cdot \cos^{2}\theta \cdot t\cdot R_{t}}{S\rho L^{2}}\cdot \left(1+2\cdot \frac{2-e^{-NL}-e^{NL}}{NL\cdot \left(e^{NL}-e^{-NL}\right)}\right)\\ N=\sqrt{\frac{S}{k_{s}tR_{t}}} \end{array}$$

**Figure 11:**
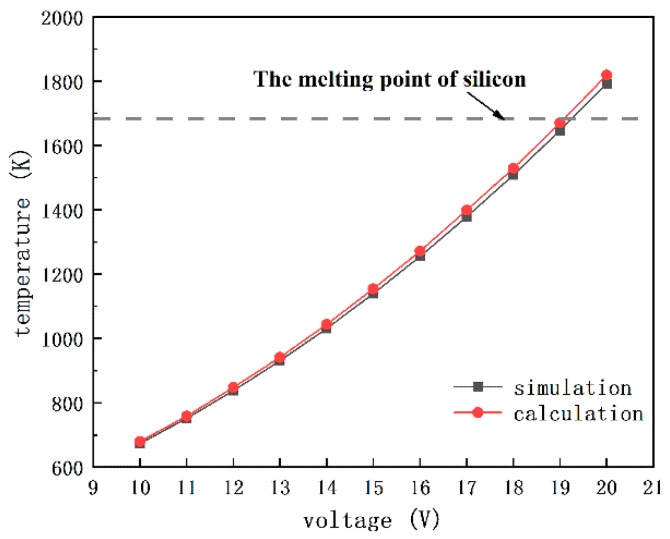
The profile of maximum temperature on the V-shape beam.

**Figure 12:**
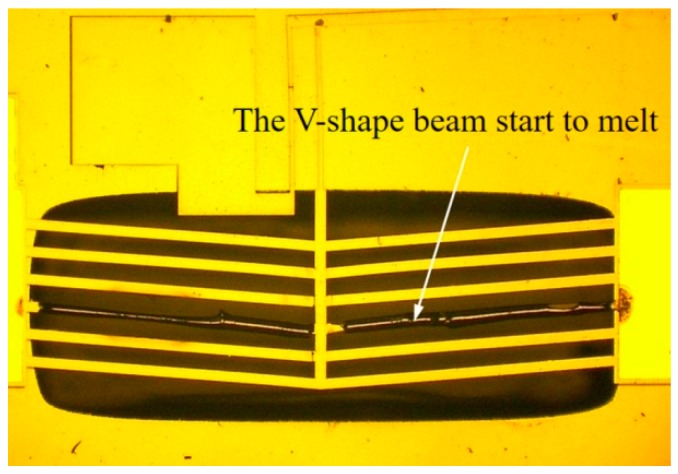
The V-shape beam start to melt with the input voltage of 19 V.

Here, $R_{t}=t_{V}/k_{V}$ represents the thermal resistivity between the gap under the bottom surface of chevron actuator. $t_{V}$ and $k_{V}$ are the thickness and the thermal conductivity coefficient of the air gap, respectively. $k_{s}$ is the thermal conductivity of silicon and ρ represents the electrical resistivity of silicon. $S=\left(2t_{V}+t+w\right)/w$ represents the shape factor that accounts for the heat transfer through all sides of the beam [19,20,21]. In addition, the finite element analysis has also been carried out to compare with the calculation. As shown in Figure 11, the maximum temperature on the V-shape beam will meet the melting point of silicon with the input voltage around 19 V, whether the calculation or the simulation, which is consistent with the test result that is shown in Figure 12.

Owing to the complementary energy method [22], P can be expressed as Equation (8).

(8) $$P=\frac{4\sin^{2}\theta \cdot AE\cos\theta }{L}\cdot \Delta y_{\max}=\frac{F_{y}}{6}$$

Combine Equation (1) with Equations (2), (6), (7), (8), and the structure parameters of the chevron actuator, the $\Delta y_{\mathrm{out}}-V$ relation can be described as:

(9) $$\{\begin{array}{c} \Delta y_{\mathrm{out}}=\left(l_{1}+l_{2}\right)\sin\frac{\left(\tau V^{2}l_{1}-F_{f}l_{3}\right)l_{f}}{EI_{f}}+\frac{\left(\tau V^{2}-F_{f}\right)l_{f}}{Etw_{f}}\\ \tau =6\cdot \frac{4\sin^{2}\theta AE\cos\theta }{L}\cdot \frac{\alpha l\sin\theta }{{\left(\frac{w}{l}\right)}^{2}+\sin^{2}\theta }\cdot \left(1+2\cdot \frac{2-e^{-NL}-e^{NL}}{NL\left(e^{NL}-e^{-NL}\right)}\right)\cdot \frac{\cos^{2}\theta tR_{t}}{S\rho L^{2}} \end{array}$$

**Figure 13:**
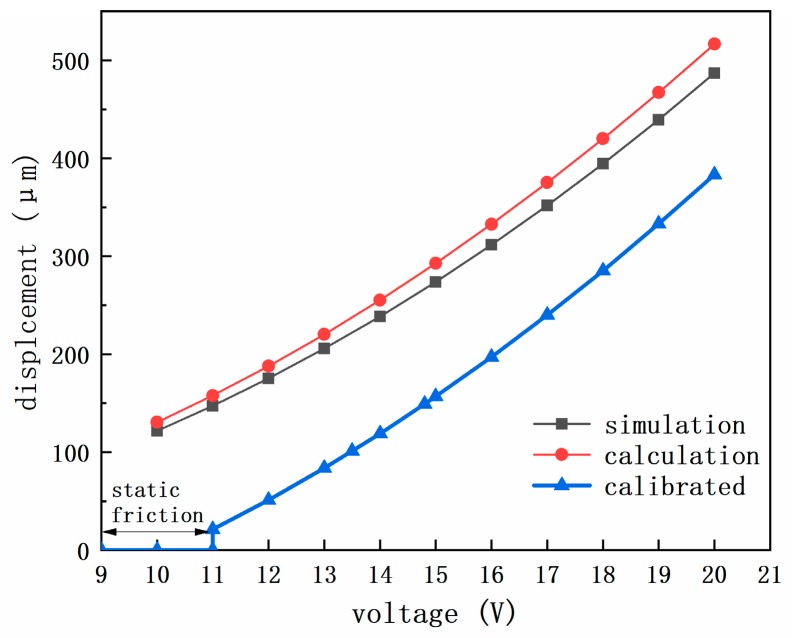
The profiles of displacement output along with input voltage.

**Figure 14:**
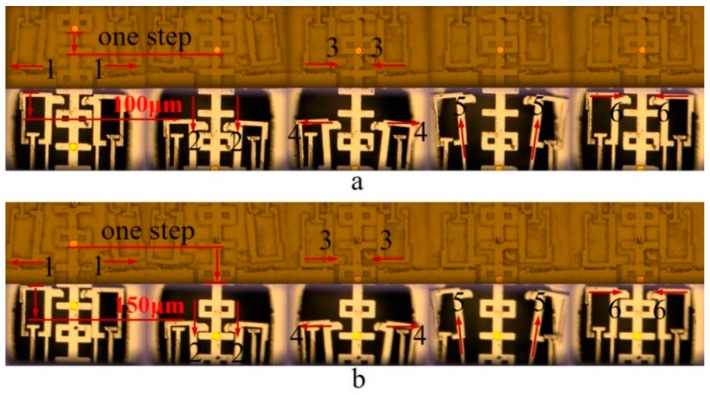
The working process of the pawls meshing with the slider. (**a**) The step with a pulling voltage of 13.5 V. (**b**) The step with a pulling voltage of 14.8 V.

Set $F_{f}=0$, $\Delta y_{\mathrm{out}}$ in the Equation (9) represents the displacement output of the lower pawl alone, without the consideration of slider pulling. The calculation and the simulation results of ANSYS (18.2) are illustrated by the profiles in Figure 13. In addition, bringing the test results, V=13.5 V/14.8 V, $\Delta y_{\mathrm{out}}=100\;\mu m/150\;\mu m$, as shown in Figure 14, back to the Equation (9), with the transcendental equation that is solved by MATLAB (2016a), we can obtain $F_{f1}=9.2\;\mathrm{mN}$, $F_{f2}=9.0\;\mathrm{mN}$, the divergence between $F_{f1}$ and $F_{f2}$ only take a percentage of 2.2%. Therefore, it’s reasonable to replace ‘$F_{f}$’ with the average number of $F_{f1}$ and $F_{f2}$. In addition, since it’s difficult to calculate the static friction directly in the present research, the effect of static friction is determined by the threshold activating voltage of the S&A chip about 11 V. And then, the profile can be calibrated as shown in Figure 13. 

The discrepancy between the calculation, simulation, and the calibrated profile reflects the effect of retarding force on the dynamic performance of the device. In terms of microtribology, the MEMS device is more influenced by surface effect [23], thus the adhesive and frictional retarding forces, which are usually comparable with forces driving device motion [24], assume a greater importance than in machines performing similar tasks at the macroscale [25].

## 4. Test and Discussion

### 4.1. The Maximum Working Voltage

The V-shape beam will start to melt, when the input voltage reaches to 19 V with a calculated maximum temperature of 1674.93 K, which is only 8.22 K minor than the melting point of silicon 1683.15 K, as shown in Figure 11. With the input voltage rising to 19.1 V, the maximum temperature $T_{\max}$ will exceed the melting point and reach to 1686.46 K. So that, it is reasonable for us to confine the working voltage below 19 V.

### 4.2. Moving Test

**Figure 15:**
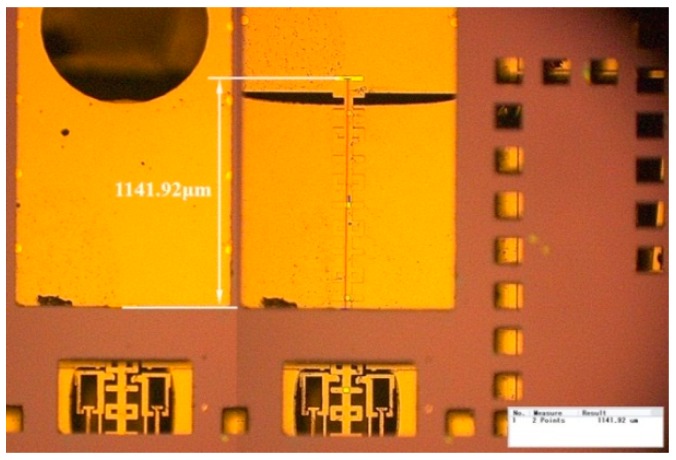
The travel range of interrupter in the S&A chip.

The working process, which corresponds with the six steps that are shown in Figure 6, is illustrated in Figure 15. We keep the substrate under the upper pawl unetched in order to make a clear distinction between the upper pawl and the lower one. Figure 14a,b, respectively, represent the process of a single step under the pulling voltage of 13.5 V and 14.8 V, with a power consumption of 3.5 W and 4.3 W.

Although the displacement outputs of the lower pawl are designed about 100 μm and 150 μm alternatively, there may be a motion discrepancy that ranges from 0 μm to 13 μm, due to the engagement gap between the pawl and slider. Meanwhile, there is not any signal input on actuator group c, so that the motion of upper pawl will be confined in horizontal direction, which makes it serve as the interlock for the decoding signal recognition.

**Figure 16:**
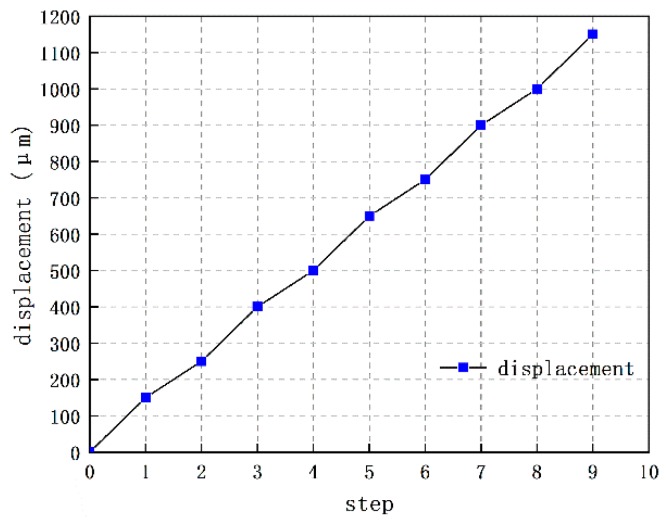
The displacement along with the steps driven by actuator.

The full range motion test of the disarming process has been carried out to verify the feasibility of our encryption system. With the series of voltage signal illustrated in Figure 8, the displacement of interrupter in the S&A chip is able to cover the range from 0 μm to 1141.92 μm, as shown in Figure 15. The disarming process is accomplished in nine steps. When compared with the travel range that we designed, 1150 μm, as shown in Figure 16, the accumulative deviation in nine steps of the disarming process, merely take a percentage of 0.7%, which seems to be acceptable for us.

### 4.3. Firing Test

In the microfuze, the high explosive pellet is detonated by the impact of a high-speed flyer, which is usually driven by another micro explosion of metal foil bridge or micro-charge. The S&A chip that is presented in this work mainly serves as a switch of the explosive train between the high explosive pellet and the micro-charge. 

**Figure 17:**
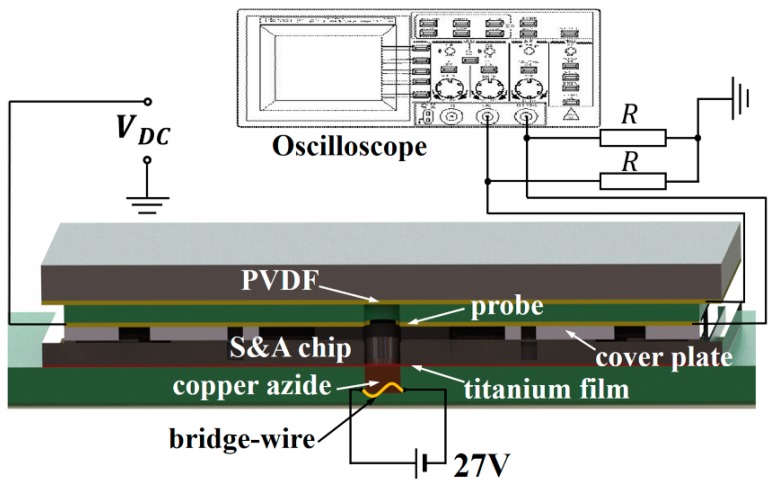
The system for the flyer velocity measurement.

Besides the function of interruption that we have instructed in the moving test, there are still some factors we should concern for the S&A chip to be applied in the microfuze, because it is rare to choose silicon as the barrel material of slapper detonator, under most circumstances it would be steel or sapphire. So that, it is reasonable to verify: if the foil between the S&A chip and the exploding chip could successfully be sheared by the edge of silicon barrel to form the flyer, and whether the acceleration of flyer could meet the threshold for the detonation of explosive. Therefore, a firing test has been carried out to measure the average velocity of the flyer coming out of the S&A chip. As shown in Figure 17, in the test system, the S&A chip assembles with a titanium film, whose thickness is 28 μm and a micro exploding chip, which consists of the copper azide that is generated by “in-situ” method, with a weight of about 0.5 mg, a bridge wire for the initiation of the copper azide, which will be activated with an input voltage of 27 V (DC). A flyer detector barrel is designed with a pair of electrode probe set at the circular inlet and the outlet covered by the PVDF (polyvinylidene fluoride) film in order to identify the average velocity.

**Figure 18:**
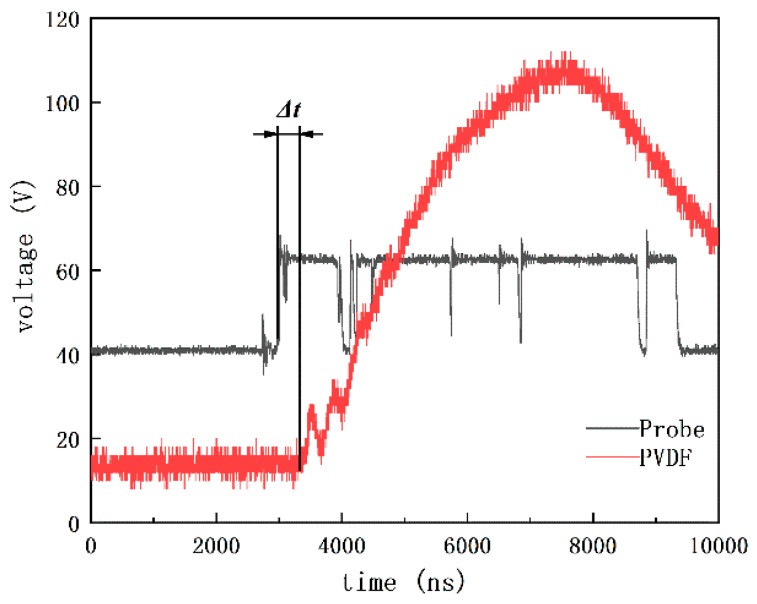
The signals detected by the probe and polyvinylidene fluoride (PVDF).

**Figure 19:**
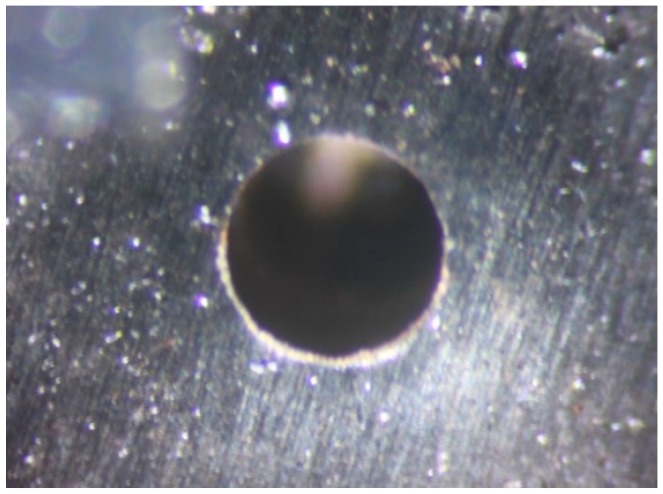
The titanium film residue with flyer sheared by the barrel.

The high temperature and high-pressure gaseous products that are generated by the explosion of copper azide will shear off and accelerate the flyer. Simultaneously, the surrounding air will be ionized and create the plasma, leading to the connection of the electrode probe, therefore the initial signal will be detected on the oscilloscope. When the highspeed titanium flyer impacts on the PVDF film, a strong piezoelectricity signal will also be transmitted to the oscilloscope, which is recognized as the end of flight. The average velocity in the detector can be obtained as *V*_avg_ *= t_d_/*Δ*t*, where Δ*t* represents the time discrepancy between the signals detected by the probe and PVDF film, as shown in Figure 18. *t_d_* is the thickness of the detector barrel. For the present research, Δ*t* = 363 ns, *t_d_* = 680 μm, so that, after a flight of about 753 μm in the SAD, the flyer is still able to reach an average velocity about *V*_avg_ *=* 1873.3 m/s. When compared with Zeng’s test results [26], the average flyer velocity and the integral circular hole on the residue of titanium film, as shown in Figure 19, indicate that there is not any obvious negative effect on the flyer acceleration that is caused by the integration of the SAD on the explosive train.

## 5. Conclusions and Perspective

In the present research, we propose a prototype of the on-chip MEMS based SAD with a mechanical encryption system.

(a) The amplitude and the timing sequence recognition of input voltages have been achieved through the meshing between the pawl and the teeth on the slider with different width.
(b) The bidirectional actuation design makes the conversion between safe status and arm status reversible. The travel range of interrupter in the SAD will only be limited by the chip dimension due to the feature of motion retention, which indicates that the design method is able to cover a wide range of microfuze with different requirement on the travel range of interrupter.
(c) The relationship between the input voltage and output of the pawl has been analyzed. Based on our design method, the prototype of encryption system can evolve into numerous kinds of variants for application in the microfuze.
(d) According to the firing test, after a flight of about 753 μm in the SAD, the titanium flyer is still able to reach an average velocity about *V*_avg_ = 1873.3 m/s, which indicates that there is not any obvious negative effect on flyer acceleration that is caused by the integration of the SAD on the explosive train.
(e) For the dynamic performance optimization, the microtribology on the interface between the slider and the substrate is worthwhile to investigate in future work.
